# Supplementary material for: Heavy resistance exercise training in older men: A responder and inter-individual variability analysis
Source: PLoS One. 2026 Jan 21;21(1):e0338775. doi: 10.1371/journal.pone.0338775 (PMC12822940; doi:10.1371/journal.pone.0338775)
Supplement: S1 Table — Key parameters of inter-individual variability for PRE to 8wk and PRE to 16wk. SDIR = √(SDLOS-EX2) – (SDLOS-SED2). Bold-phase indicates when SDLOS-EX > SD LOS-SED, indicating training induced interindividual variability. * Indicate when SDIR exceeds the TE. Abbreviations: TE, typical error; SDLOS-SED, SD of losartan with sedentary; SDLOS-EX, SD of losartan with exercise; SDIR, SD of individual responses; Nm, newton meter; qCSA, quadriceps cross-sectional area; fCSA, fibre cross-sectional area. (PPTX) [file pone.0338775.s001.pptx]

## Slide 1
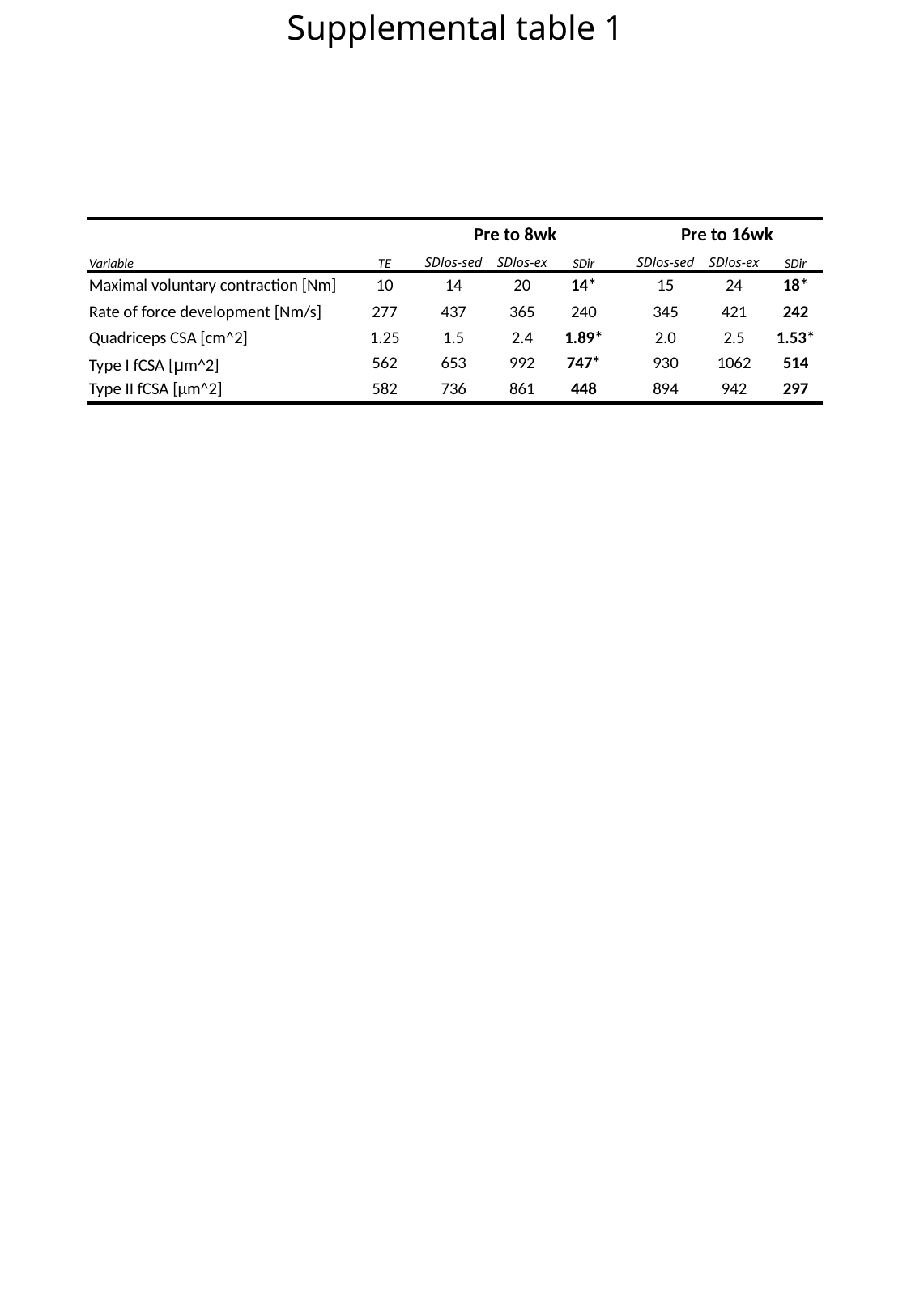

Supplemental table 1
| | | Pre to 8wk | | | | Pre to 16wk | | |
| --- | --- | --- | --- | --- | --- | --- | --- | --- |
| Variable | TE | SDlos-sed | SDlos-ex | SDir | | SDlos-sed | SDlos-ex | SDir |
| Maximal voluntary contraction [Nm] | 10 | 14 | 20 | 14\* | | 15 | 24 | 18\* |
| Rate of force development [Nm/s] | 277 | 437 | 365 | 240 | | 345 | 421 | 242 |
| Quadriceps CSA [cm^2] | 1.25 | 1.5 | 2.4 | 1.89\* | | 2.0 | 2.5 | 1.53\* |
| Type I fCSA [μm^2] | 562 | 653 | 992 | 747\* | | 930 | 1062 | 514 |
| Type II fCSA [μm^2] | 582 | 736 | 861 | 448 | | 894 | 942 | 297 |
