# Supplementary material for: Heavy resistance exercise training in older men: A responder and inter-individual variability analysis
Source: PLoS One. 2026 Jan 21;21(1):e0338775. doi: 10.1371/journal.pone.0338775 (PMC12822940; doi:10.1371/journal.pone.0338775)
Supplement: S2 Table — Changes at the group level at PRE to 8wk (top) and PRE to 16wk (bottom) shown as percent change, delta values, and effect sizes. Data are means ± SD except effect sizes which are show with 95% CI. Abbreviations: PLA-EX, Placebo Exercise; LOS-EX, Losartan Exercise; ES, Effect Size; Nm, newton meter; qCSA, quadriceps cross-sectional area; fCSA, fibre cross-sectional area. (PPTX) [file pone.0338775.s002.pptx]

## Slide 1
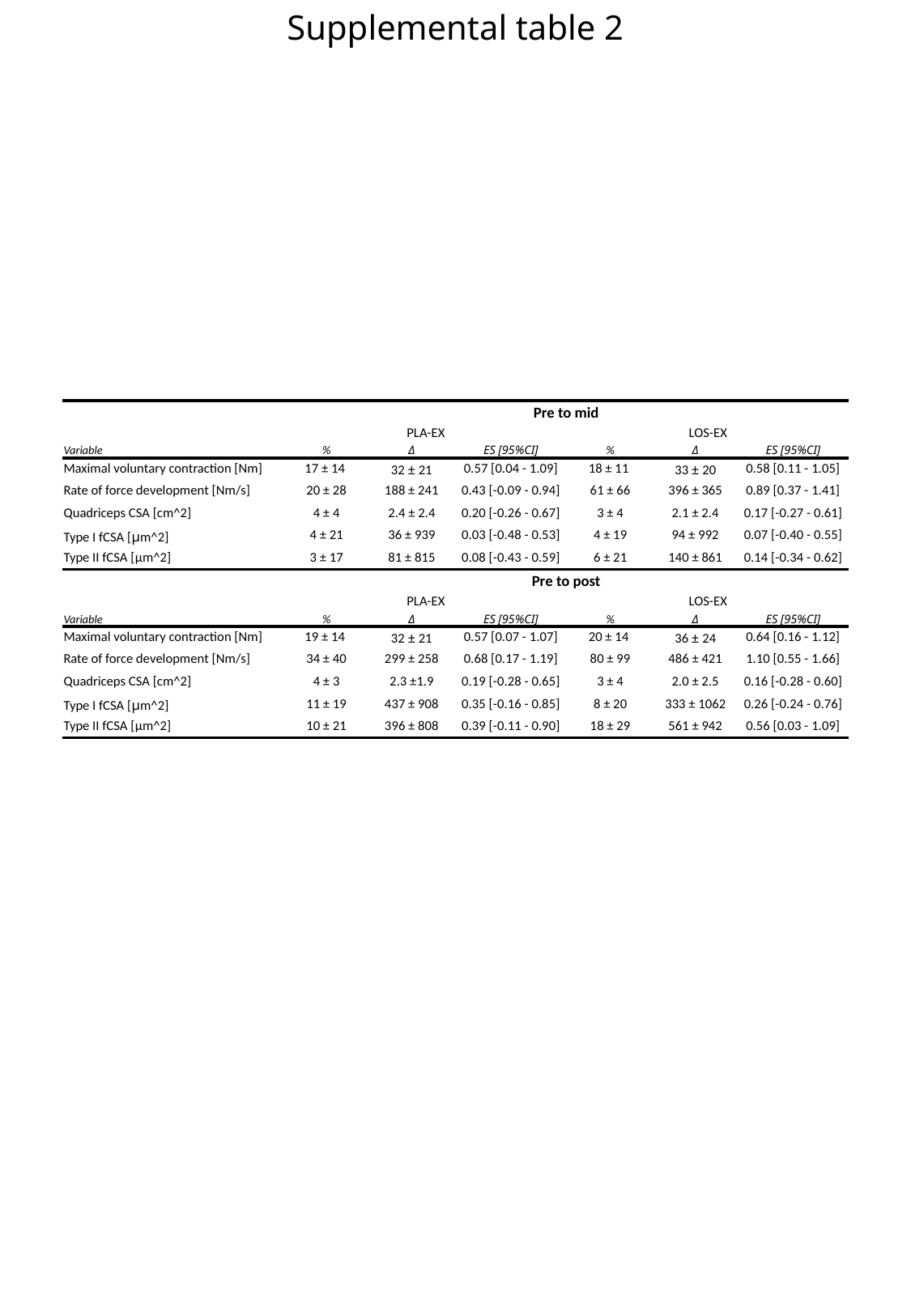

Supplemental table 2
| | Pre to mid | | | | | |
| --- | --- | --- | --- | --- | --- | --- |
| | PLA-EX | | | LOS-EX | | |
| Variable | % | Δ | ES [95%CI] | % | Δ | ES [95%CI] |
| Maximal voluntary contraction [Nm] | 17 ± 14 | 32 ± 21 | 0.57 [0.04 - 1.09] | 18 ± 11 | 33 ± 20 | 0.58 [0.11 - 1.05] |
| Rate of force development [Nm/s] | 20 ± 28 | 188 ± 241 | 0.43 [-0.09 - 0.94] | 61 ± 66 | 396 ± 365 | 0.89 [0.37 - 1.41] |
| Quadriceps CSA [cm^2] | 4 ± 4 | 2.4 ± 2.4 | 0.20 [-0.26 - 0.67] | 3 ± 4 | 2.1 ± 2.4 | 0.17 [-0.27 - 0.61] |
| Type I fCSA [μm^2] | 4 ± 21 | 36 ± 939 | 0.03 [-0.48 - 0.53] | 4 ± 19 | 94 ± 992 | 0.07 [-0.40 - 0.55] |
| Type II fCSA [μm^2] | 3 ± 17 | 81 ± 815 | 0.08 [-0.43 - 0.59] | 6 ± 21 | 140 ± 861 | 0.14 [-0.34 - 0.62] |
| | Pre to post | | | | | |
| | PLA-EX | | | LOS-EX | | |
| Variable | % | Δ | ES [95%CI] | % | Δ | ES [95%CI] |
| Maximal voluntary contraction [Nm] | 19 ± 14 | 32 ± 21 | 0.57 [0.07 - 1.07] | 20 ± 14 | 36 ± 24 | 0.64 [0.16 - 1.12] |
| Rate of force development [Nm/s] | 34 ± 40 | 299 ± 258 | 0.68 [0.17 - 1.19] | 80 ± 99 | 486 ± 421 | 1.10 [0.55 - 1.66] |
| Quadriceps CSA [cm^2] | 4 ± 3 | 2.3 ±1.9 | 0.19 [-0.28 - 0.65] | 3 ± 4 | 2.0 ± 2.5 | 0.16 [-0.28 - 0.60] |
| Type I fCSA [μm^2] | 11 ± 19 | 437 ± 908 | 0.35 [-0.16 - 0.85] | 8 ± 20 | 333 ± 1062 | 0.26 [-0.24 - 0.76] |
| Type II fCSA [μm^2] | 10 ± 21 | 396 ± 808 | 0.39 [-0.11 - 0.90] | 18 ± 29 | 561 ± 942 | 0.56 [0.03 - 1.09] |
