# Supplementary material for: Heavy resistance exercise training in older men: A responder and inter-individual variability analysis
Source: PLoS One. 2026 Jan 21;21(1):e0338775. doi: 10.1371/journal.pone.0338775 (PMC12822940; doi:10.1371/journal.pone.0338775)
Supplement: Attachment — Submitted filename: Rebuttal.docx [file pone.0338775.s007.docx]

**Reviewer 1**

The authors found that there is a large degree of inter-individual differences in response to HReT in older males and that non-responders are rare/non-existent. They conclude that HReT should be a universally recommended strategy for improving muscle mass/strength. The authors looked at group effects, as well as individual effects and assessed inter-individual variability by statistically comparing the differences in standard deviations between the control and exercise groups. The research question is novel and is addressed by the methods employed by the authors. Overall, the paper is well-written; however, I have some concerns and comments below to be addressed.
We sincerely thank the reviewer for the comprehensive and structured evaluation of our manuscript, as well as for the positive comments regarding its novelty, clarity, and methodological approach. We have carefully considered each of the points raised and revised the manuscript accordingly to address them to the best of our ability.

Comments:
• Please run statistics on the participant characteristics and report this in the methods, as well as in Table 1.

We have now performed statistical comparisons of baseline participant characteristics between groups using independent samples t-tests. The corresponding p-values have been added to Table 1, and the statistical procedure is described in the Methods section: “Baseline participant characteristics were compared between groups using unpaired two-tailed t-tests.”

• Please add individual datapoints to Figure 3.

Individual data points have been added to Figure 3.

• The physical activity of the population recruited is classified as “not performing regular strenuous exercise.” Training status is a well-identified confounder of training response. The authors should provide a more detailed explanation of this inclusion criteria (i.e., how was “strenuous” classified, and how was “regular” classified) so that the initial training status of the volunteers is clearer. The authors should also discuss the degree that differences in initial training status may have impacted the inter-individual differences demonstrated.

We agree that training status is an important determinant of training responsiveness and have clarified the inclusion criteria in the Methods. Participants were classified as non-exercising based on self-reported training history. Specifically, they were sedentary or moderately active, performing no structured strength training or other regular strenuous exercise on a regular basis, except for activities such as walking or cycling as transportation. This corresponds to Tier 0–1 in the framework proposed by McKay et al. (2022) [1].

We have added the following to method: “Potential participants had to be sedentary or moderately active, performing no structured strength training or other regular strenuous exercise on a daily or weekly basis, except for activities such as walking or cycling as transportation. This corresponds to Tier 0–1 in the framework proposed by McKay et al., 2022.”

We have further added a sentence in the discussion to reflect this point: “It should also be pointed out that, although prior training history has been suggested to modulate subsequent responsiveness to exercise interventions [2], all participants in the present study were naïve to HReT prior to enrollment”

• The strength data presented in Figure 4 is presented in absolute terms, rather than normalized to bodyweight or muscle mass. Given that body size largely confounds absolute measures of muscle strength (and bodyweight varied greatly between the participants of this study as the range was reported as 62-102kg), c? Also, it is mentioned in the discussion that initial strength correlated negatively with strength improvements, which may give the false assumption that the individuals with lower initial strength are less trained; however, we don’t know this for certain, as they may have been the participants of smaller body size. Can the authors please clarify the significance of this correlation when it is being made in absolute terms and explain further why these differences may exist?

We agree that normalization of strength data can be relevant in certain contexts, particularly when comparing groups that differ in body size or composition. However, in this study, normalizing to total body weight could introduce additional confounding, as body weight includes fat mass, which is not directly relevant to force production. Given that all participants were older men from a single study cohort and that our primary analyses focused on training-induced changes within individuals, we considered absolute values to be the most appropriate representation of the outcomes. It should also be noted that absolute strength values are commonly reported in similar studies [3–5].

For transparency, we have now provided the data as part of this reply (which will be available online) both in absolute terms and normalized to baseline body weight. As the reviewer rightly notes, body weight does appear to contribute to baseline strength differences; however, the negative association between baseline strength and relative change remains when strength is normalized, indicating that this pattern cannot be explained solely by body size.

To further clarify this point, we have updated the Discussion to read: “Lastly, we observed that individuals with lower baseline values in MVC (**also when normalized to bodyweight**), RFD, and type II fCSA”

Finally, we emphasize that the negative correlation between baseline strength and training-induced improvements likely reflects a combination of ceiling effects and regression to the mean, rather than differences in body size or training status. This is now explicitly stated in the Discussion: “While this pattern likely reflects, at least in part, true biological differences in adaptive capacity, it may also be influenced by ceiling effects and the statistical phenomenon of regression to the mean, which can exaggerate the appearance of greater gains among those with lower baseline values.”

• Given that sets, %1RM and reps differed between participants, is it possible that differences in volume accounted for some of the inter-individual differences? If volume can be calculated from exercise logs, this could be a nice addition to the investigation.

We appreciate the reviewer’s comment and the opportunity to clarify this point. All participants followed the exact same structured training program and progression, with identical prescribed sets, repetitions, and relative training intensities across all phases. Consequently, there were no systematic differences in training volume between participants. Minor variations may have occurred due to natural fluctuations in load adjustments on a session-to-session basis to maintain high exertion, but these do not represent individualized training volumes.

To make this clearer compared to the previous version, the description of the training intervention has been expanded and now reads: “The intervention lasted 16 weeks, and participants were tested before (PRE), midway (8wk) and after (16wk). Participants randomised into EX exercised thrice weekly for 16 weeks (48 scheduled sessions). At each session, participants performed three lower body exercises (seated leg extension, horizontal leg press, and seated leg curl) and two upper body exercises (pulldown and machine shoulder press). For the leg press and leg extension exercises, the training program consisted of six distinct phases that systematically increased training intensity and reduced the number of repetitions.

- Leg press: 3 × 12 at 15 RM (Phase 1), 4 × 10 at 12 RM (Phase 2), 5 × 8-10 at 10 RM (Phase 3), 5 × 6–10 at 8-10 RM (Phase 4), 4 × 6–8 at 8 RM (Phase 5), and 4 × 4–8 at 6-8 RM (Phase 6).
- Leg extension: 3 × 12 at 15 RM (Phase 1), 4 × 10 at 12 RM (Phase 2), 4 × 10 at 10 RM (Phase 3), 5 × 8–10 at 10 RM (Phase 4), 5 × 6–8 at 8 RM (Phase 5), and 4 × 6–8 at 8 RM (Phase 6).

The leg curl followed a similar structured progression but with slightly fewer sets. Further details on the training program are available elsewhere (Heisterberg et al., 2018). The 1-repetition maximum (i.e. the heaviest load that can be lifted once) in leg press, leg extension, and leg curl was evaluated before session number 1, 7, 16, 25, 34 and 43. The load used during training was based on the prior 1RM result, although the load was adjusted on a session-by-session basis to secure a high degree of exertion, defined as performing repetitions until concentric failure (inability to complete another repetition with proper technique). All sessions were supervised by study personnel, who also logged weight used and number of repetitions performed for each exercise at each session. “

• Line 391-393: Please add these calculations to the supplementary file.
We thank the reviewer for this suggestion. The SDIR calculations were performed using the same statistical procedure already described in the Methods section. Because no additional or unique analytical steps were applied beyond what is already described, we have chosen not to include the raw calculations in the supplementary materials to maintain consistency with how the main analyses are presented.

We do however here provide the calculations as part of the response.

**8 week**

- MVC: SQRT((19.6^2) - (14.0)) = 14
- RFD: SQRT((437^2) - (365)) = 240 (SED and EX reversed)
- qCSA: SQRT((2.42^2) - (1.52)) = 1.9
- Type I fCSA: SQRT((992^2) - (653)) = 747
- Type II fCSA: SQRT((861^2) - (736)) = 448

**16 week**

- MVC: SQRT((23.8^2) - (15.0)) = 18
- RFD: SQRT((421^2) - (345)) = 242
- qCSA: SQRT((2.54^2) - (2.0)) = 1.5
- Type I fCSA: SQRT((1062^2) - (930)) = 514
- Type II fCSA: SQRT((942^2) - (894)) = 297

• Line 30: for clarity, it would be helpful to the reader if it were clear that the values provided in the abstract are changes after 16 weeks.

Amended.

• Line 31: value of 82% does not match the value reported in the results section (which was 81%).

The small discrepancy reflects rounding procedures. The proportions at 16 weeks were 42.11% (Robust) and 39.47% (Excellent). When reported separately, each value was rounded down (42% and 39%), whereas their combined value (81.58%) was rounded up to 82%, in accordance with standard rounding rules. We therefore prefer to retain the reported values of 42%, 39%, and 82%, as this is mathematically consistent and does not affect interpretation.

• Line 106: not performing? regular strenuous exercise

Amended.

• Line 122: (15–6)?

• Line 122: Please clarify why the number of sets differed and what determined this difference.

The training program section has been re-written to provide more detail. Importantly, all participants followed the same structured training program, which we have clarified by writing explicitly the number of sets for each of the two main lower body exercises. The revised text now reads: “For the leg press and leg extension exercises, the training program consisted of six distinct phases that systematically increased training intensity and reduced the number of repetitions.

• Leg press: 3 × 12 at 15 RM (Phase 1), 4 × 10 at 12 RM (Phase 2), 5 × 8-10 at 10 RM (Phase 3), 5 × 6–10 at 8-10 RM (Phase 4), 4 × 6–8 at 8 RM (Phase 5), and 4 × 4–8 at 6-8 RM (Phase 6).

• Leg extension: 3 × 12 at 15 RM (Phase 1), 4 × 10 at 12 RM (Phase 2), 4 × 10 at 10 RM (Phase 3), 5 × 8–10 at 10 RM (Phase 4), 5 × 6–8 at 8 RM (Phase 5), and 4 × 6–8 at 8 RM (Phase 6).

The leg curl followed a similar structured progression but with slightly fewer sets. Further details on the training program are available elsewhere.”

• Line 125: Please clarify how a “high degree of exertion” was indicated.

We have clarified this in the text. Specifically, a high degree of exertion refers to performing repetitions until concentric (positive) failure, defined as the inability to complete another repetition with proper technique. The revised sentence now reads: “The load used during training was based on the prior 1RM result, although the load was adjusted on a session-by-session basis to secure a high degree of exertion, defined as performing repetitions until concentric failure (inability to complete another repetition with proper technique).”

• Section 2.5.1: Please indicate how many MVC trials were run and how participants were instructed to accurately measure RFD (i.e., were they instructed to “kick as hard and as quickly as possible” during each trial?)
We have now specified the number of MVC trials and participant instructions in the manuscript. Each participant performed three MVC attempts, and participants were instructed to contract “as hard and as fast as possible”. We have added the following: “Participants performed three MVC attempts and were instructed to contract “as hard and as fast as possible”.

Section 2.5.3
o Please indicate which muscle the biopsy was taken from and the technique used (i.e., Bergström, punch biopsy).
o Please indicate how the muscle was preserved (i.e., flash frozen, embedded in paraffin) and sectioned (i.e., cryostat, microtome).
o Please provide additional details on the fibre-typing stain employed (i.e., incubation times, buffers used).
o Please provide additional details on image acquisition and how the analysis was conducted.

We have expanded the description of the biopsy procedure, sample handling, staining, and analysis to provide the requested detail. The revised text now reads: ” A total of three muscle biopsies were obtained, using Bergström needles with manual suction (Bergstrom, 1975), from the vastus lateralis muscle of each individual; one at each time point. The samples at PRE and 16wk were taken from the same leg, through different incision sites, 3 cm apart. The sample at 8wk was taken from the contralateral leg. Pieces of muscle tissue were embedded in OCT compound (Tissue-Tek; Sakura Finetek Europe, Alphenaan den Rijn, The Netherlands), and frozen in isopentane (2-Methylbutan; J. T. Baker, Avantor Performance Materials, Deventer, The Netherland) pre-cooled in liquid nitrogen. Samples were stored at −80 °C until further processing. Cross-sections (10 µm) were cut in a cryostat and subjected to ATPase staining at pH 4.37, 4.53, 4.57, and 10.30 to differentiate type I and type II fibers. Stained sections were imaged using a light microscope (Olympus BX40 microscope (Olympus Optical, Tokyo, Japan)), and the borders of individual fibers were manually outlined for calculation of fiber type–specific cross-sectional area (fCSA). The same person analysed all samples, blinded to group and time. fCSA has been published elsewhere as group means (Heisterberg et al., 2018; Soendenbroe et al., 2022).”

• Line 159: Was a true 1RM measured for each participant, or was a multi-repetition max sometimes used? If a multi-rep max was used, please clarify this in the methods.

A true 1RM was measured for all participants at all time points.

• Line 208: Please clarify if significance was accepted at < or ≤ 0.05.

< 0.05. Specified.

• Table 1: Please define all abbreviations in the table legend.

Amended.

• Table 2: Please add effect size (ES) abbreviation definition to the table legend.

Amended.

• Line 241: Please provide an explanation for the missing data demonstrated in Figure 1.

• Line 247: Please provide an explanation for the missing data demonstrated in Figure 1.

MVC and RFD were derived from the same test. In the EX group, 8-week data are missing for two participants (21 and 35). For one participant, the data were mistakenly overwritten and thereby lost. For the other, no information is available on why the data are missing. These missing data points are consistent with the dataset reported in previous publications [6,7]. In the SED group, baseline data are missing for one participant, which precluded calculation of Δ and % change values at 8 and 16 weeks. The reason for this missing data is unclear, but this is likewise consistent with previous publications. This has been specified in result section: “For MVC/RFD, analyses were performed on 36/38 participants in EX and 19/20 in SED at baseline and 8wk, and on 38/38 and 19/20, respectively, at baseline and 16wk.”

• Line 253: Please provide an explanation for the missing data demonstrated in Figure 1.

There were no missing data for qCSA. All available participants were included in the analyses (n = 38 in EX and n = 20 in SED). This has been specified in result section:” For qCSA, analyses were performed on all participants (38/38 in EX and 20/20 in SED) at baseline, 8wk, and 16wk.”

• Line 259: Please provide an explanation for the missing data demonstrated in Figure 1.

For fCSA, in the EX group, 32 out of 38 participants had biopsy material of sufficient quality (e.g., ) at both baseline and 8 weeks. Similarly, 32 out of 38 participants (not the exact same 32) had material of sufficient quality at baseline and 16 weeks. In the SED group, the corresponding numbers were 18 out of 20 at baseline and 8 weeks, and 17 out of 20 at baseline and 16 weeks. Loss of data in this type of biopsy analysis is common and typically reflects limitations in sample quality or insufficient fiber yield for reliable quantification [8]. The average number of fiber used for measure fiber CSA was 140±2.

This has been specified in result section: “Analyses were performed on 32/38 participants in EX and 18/20 in SED at baseline and 8wk, and on 32/38 and 17/20, respectively, at baseline and 16wks.”

• Line 336: See comment regarding Line 31.

The small discrepancy reflects rounding procedures. The proportions at 16 weeks were 42.11% (Robust) and 39.47% (Excellent). When reported separately, each value was rounded down (42% and 39%), whereas their combined value (81.58%) was rounded up to 82%, in accordance with standard rounding rules. We therefore prefer to retain the reported values of 42%, 39%, and 82%, as this is mathematically consistent and does not affect interpretation.

• Line 345-348: Given that neural adaptation to resistance exercise typically precedes structural adaptation (i.e., hypertrophy), it may provide value to comment on this point and the nuance of classifying non/poor-responders (i.e., should strength or muscle size be the key metric? Which is more important for health outcomes?).

We fully agree with this point and have added the following text to the Discussion: -

- “Neural adaptations, such as improved motor unit recruitment and firing efficiency, typically manifest earlier, whereas hypertrophic processes begin from the first training sessions but only become detectable later.”
- “Given that nearly all participants demonstrated early strength gains, responder classification after 8 weeks largely depends on whether these functional improvements were accompanied by measurable hypertrophy, which typically develops on a slower time course.”

Importantly, both muscle mass and strength are strongly associated with health outcomes, and likely provide complementary perspectives.

• Line 396: “at” the group level.

Amended.

• Line 457: For clarity of readership, it might be helpful to indicate that the data presented in Figure 4 are from the exercising group only. Stating data is from “all participants” may be misleading.

Amended.

**Reviewer 2**

The paper “Heavy Resistance Exercise Training in Older Men: A Responder and Inter-individual Variability Analysis” by Soendenbroe et al. examines the variability in skeletal muscle adaptations among older men following 16 weeks of supervised heavy resistance exercise training (HReT). Fifty-eight healthy men aged approximately 72 years were randomized to an exercise (n = 38) or sedentary control (n = 20) group. Strength and muscle morphology were assessed through maximal voluntary contraction (MVC), rate of force development (RFD), quadriceps cross-sectional area (qCSA), and fibre cross-sectional area (fCSA) of type I and II fibres. Using the standard deviation of individual responses (SDIR) and classification based on changes exceeding the typical error, the authors quantified inter-individual variability and categorized participants as Poor, Trivial, Robust, or Excellent responders.

The study directly addresses a long-standing debate in exercise physiology regarding the prevalence of “non-responders.” By applying a rigorous statistical framework that distinguishes between biological variation and measurement error, and genuine inter-individual differences, the authors advance the field beyond simple descriptive interpretations of variability. The findings support a reassuringly optimistic message for clinicians and policy makers: virtually all older adults benefit meaningfully from structured resistance exercise.

The use of a randomized controlled design, inclusion of a non-exercising comparator, and the integration of multiple gold-standard outcome measures (MRI-derived qCSA, histological fCSA, and dynamometry-based strength) strengthen the validity of the conclusions. The authors’ application of SDIR and typical error–based classification is statistically transparent and aligns with best practices recently advocated in the literature (e.g., Atkinson & Batterham, 2015; Bonafiglia et al., 2021). Moreover, the two-pronged approach, quantifying variability globally and then examining individual trajectories, provides a nuanced understanding that is rarely achieved in training studies.

We sincerely appreciate the reviewer’s thoughtful and encouraging assessment of our study. We have carefully considered all specific comments and suggestions and revised the manuscript accordingly to strengthen clarity, transparency, and methodological detail.

However, several limitations warrant attention. The sample size, although respectable, limits the power to detect small moderating effects and inflates uncertainty in the estimation of variability. Pooling participants from the losartan and placebo arms of a prior trial could introduce residual confounding, even if sensitivity analyses suggested no drug effect. The study focuses exclusively on older men, which constrains generalizability to women and to frailer or multimorbid populations who may respond differently to loading stimuli. The analysis assumes homogeneity of measurement error between groups and across time points, an assumption that may not hold given the variability of biopsy and MRI results. Furthermore, although the responder classification framework is rigorous, the arbitrary thresholds for categorizing “Robust” versus “Excellent” responders may exaggerate apparent distinctions. It is also notable that the physiological mechanisms underpinning variability, such as neural drive, muscle fibre type distribution, or molecular signalling, were not explored, which limits the ability to explain the observed heterogeneity. Some attention to these issues is warranted.

We fully agree with the reviewer’s insightful comments regarding the study’s limitations and the absence of mechanistic follow-up work. To address this, we have added a dedicated Limitations section to the Discussion, which explicitly details:

1. Sample size.
2. Pooling of participants.
3. Inclusion of only healthy older men.
4. The assumption of homogeneity of measurement error.
5. The use of an arbitrary thresholding approach to classify responders.
6. The absence of mechanistic investigations explaining variability in response.

The section reads: “This study has several limitations that should be considered when interpreting the findings. First, the sample size was modest (n = 38 in the exercise group and n = 20 in the control group), which may limit statistical power to detect subtle effects. Second, the analysis pooled participants who received either losartan or placebo. Although previous work from our group has shown no effect of losartan on muscle maintenance or neural innervation [6,7], we re-calculated SD_IR_ values including only participants who received losartan, and the results were unchanged. Nonetheless, we cannot fully exclude the possibility that subtle effects were not detected. Third, the study did not include a true control group without either exercise or losartan treatment, which may have limited the ability to fully isolate training effects from other influences. Fourth, the study included only healthy older men, and no women or frail individuals were recruited. This homogeneity increases internal validity but limits the generalizability of the findings to broader populations. Fifth, our analytical framework assumes homogeneity of measurement error across groups and time points, an assumption that was not tested. Sixth, the classification of individuals into Poor, Trivial, Robust, and Excellent responders was based on a specific thresholding approach (typical error). While widely used, this approach is inherently arbitrary, and different thresholds or statistical frameworks (e.g., smallest worthwhile change) could yield different classifications. Finally, the present study did not include mechanistic investigations aimed at explaining *why* inter-individual variability occurs. Future work should aim to integrate mechanistic endpoints to help delineate the biological drivers underlying differential training responsiveness.”

While addressing these issues - particularly mechanistic investigations - would indeed strengthen the work, we believe the present findings are of substantial value as the first to address the existence of true inter-individual variability in response to heavy resistance exercise training in older men using a rigorous statistical framework. Our intention is that this framework can serve as a foundation for future studies designed to address precisely these questions: Why do some individuals respond more robustly than others? Is there a small subset with truly limited responsiveness, and what mechanisms explain these differences?

The discussion effectively contextualizes the findings within the existing literature, contrasting the study with reports of non-responders in both young and older cohorts. The authors’ avoidance of the term “non-responder” is commendable, as most participants improved in at least one outcome. Nevertheless, some claims verge on over-interpretation; concluding that “true non-responders are rare” may not be fully supported, given the modest sample and lack of replication. Additionally, functional outcomes relevant to older adults (e.g., gait speed, chair-rise performance) and quality-of-life-related measures were not included, which would have enhanced clinical translation.

To avoid over-interpretation, we have revised the wording in both the Abstract and Discussion to clarify that the rarity of non-responders refers specifically to this population of healthy older men, rather than making a generalizable statement. The revised text now reads:

- Abstract conclusion: “Given the rarity of true non-responders, **our data support** HReT as the universally recommended first-line strategy for enhancing muscle mass and strength”
- Discussion: “Given the rarity - or possible absence - of true non-responders **in this population**, future research should prioritize optimizing training strategies broadly and improving the accessibility of HReT, …”

In addition, we have added a statement in the Discussion acknowledging that clinically relevant outcomes (e.g., physical performance, activities of daily living, and quality of life) were not assessed in this study, which limits direct clinical translation. This reads: “Moreover, future studies should incorporate clinically relevant outcomes such as physical performance, activities of daily living, and quality of life, particularly in frail older adults, to strengthen the translational impact of these findings.”

The authors correctly note that baseline strength inversely predicts relative gain, consistent with regression-to-the-mean phenomena. Nonetheless, their framing that HReT should remain a universal prescription is not directly supported by the study’s limited sample and lack of functional outcome measures (e.g., gait speed, balance).

We thank the reviewer for this thoughtful and constructive comment. In response, we have implemented several revisions to address these concerns:

- Added a Limitations section explicitly acknowledging the modest sample size and how this may limit the generalizability of our findings.
- Added a sentence in the Discussion highlighting that clinically oriented outcomes (e.g., physical performance, activities of daily living, and quality of life) were not assessed in this study.
- Included a sentence on regression to the mean in the Discussion to contextualize the relationship between baseline strength and relative improvements: “While this pattern likely reflects, at least in part, true biological differences in adaptive capacity, it may also be influenced by ceiling effects and the statistical phenomenon of regression to the mean, which can exaggerate the appearance of greater gains among those with lower baseline values.”
- Revised several statements throughout the manuscript to clarify that the conclusion regarding the benefits of HReT applies specifically to this population of healthy older men, rather than to older adults more broadly.

We believe these changes address the reviewer’s concerns and provide a more balanced interpretation of the study’s implications.

The presentation of individual data (Figures 1–2) is excellent and transparent, aligning with open-science practices. However, the TE derivation relies on pooled SED data across both 8- and 16-week intervals, which may conflate temporal variability. The authors’ responder categorization (Poor, Trivial, Robust, Excellent) is heuristic rather than validated, and no sensitivity analysis using alternative thresholds (e.g., smallest worthwhile change) was shown. Furthermore, conclusions about the rarity of non-responders may be overstated, given the wide confidence intervals for individual effects and potential measurement artifacts (as acknowledged for one apparent case of muscle loss).

We acknowledge that the typical error (TE) was calculated by pooling data across both 8- and 16-week intervals. This was an intentional decision to ensure a single, consistent TE threshold for each outcome, thereby avoiding situations where an identical change might classify as a “response” at one time point but not at another purely due to differing error estimates. Given that participants entered the study on a rolling basis over the course of a year - with baseline, 8-week, and 16-week assessments intermingled - we considered this pooling strategy to be the most appropriate and balanced solution. We have clarified this rationale in the Methods section: “TE was derived from pooled SED data across both 8- and 16-week intervals. This approach was chosen to ensure a single, consistent threshold for defining individual responsiveness across time points, which we deemed appropriate given the rolling inclusion of participants and overlapping assessments”.

Regarding response classification, we agree that the Poor / Trivial / Robust / Excellent categories were chosen primarily for their clarity and ease of interpretation. These thresholds were not derived from a gold standard or evidence-based clinical cut-offs but reflect a pragmatic approach commonly used in exercise physiology, where individual responses are interpreted relative to the typical error (TE). While alternative thresholds, such as the smallest worthwhile change (SWC), could also be applied, our aim was not to validate a specific classification system but to provide an accessible illustration of the distribution of individual responses. We have added a note in the Discussion acknowledging this and suggesting SWC and related approaches as complementary strategies: “In the present study, only TE was applied as the response threshold; however, alternative thresholds such as SWC could be used in future analyses to explore the robustness of these classifications.“

Regarding confidence intervals, we also agree that the confidence intervals surrounding individual estimates are wide for some outcomes. In our view, this highlights an important point. Despite a highly standardized and tightly supervised intervention - where every set of every exercise session was logged, and gold-standard measurement techniques were used - substantial inter-individual variability persisted.

Providing confidence intervals for SDIR values in Table 3 could further strengthen the quantitative interpretation.

We agree with the reviewer that providing confidence intervals strengthens the quantitative interpretation of the SDIR values. Confidence intervals for SDIR have been added to Table 3. In addition, a brief description of how these confidence intervals were calculated has been added to the Methods section to improve transparency and reproducibility

The discussion could expand on potential mechanistic correlates (e.g., neural vs hypertrophic contributions) to individual variability.

We agree with the reviewer and have expanded the Discussion with the following statement:

- “Neural adaptations, such as improved motor unit recruitment and firing efficiency, typically manifest earlier, whereas hypertrophic processes begin from the first training sessions but only become detectable later.”
- “Given that nearly all participants demonstrated early strength gains, responder classification after 8 weeks largely depends on whether these functional improvements were accompanied by measurable hypertrophy, which typically develops on a slower time course”

**Reviewer 3**

This manuscript addresses an important topic in the field of exercise physiology by investigating inter-individual variability in muscle adaptations to resistance training among older adults. The study applies a comprehensive analytical framework and presents individual-level data in a transparent and methodologically grounded manner. Nonetheless, several methodological and interpretative limitations should be considered to contextualize the findings and improve the clarity, robustness, and generalizability of the conclusions.

We appreciate the reviewer taking the time to evaluate our work and provide thoughtful feedback. We have carefully considered each of the comments and have revised the manuscript where appropriate to clarify methodological details and strengthen the interpretation of our findings.

1. Introduction
Given that this is a secondary analysis, it would be helpful if the authors provided a brief description of the original study design, including sample characteristics, intervention duration, and training protocol. Including this information, particularly in the third paragraph, where the aims and methodological approach are introduced, would improve the clarity and contextualization of the study. Clearly stating the origin and nature of the dataset would also help readers better assess the scope and scientific contribution of the present analysis.

We agree with the reviewer that a brief description of the original study design strengthens the clarity and contextualization of the manuscript. We have therefore added a sentence in the Introduction specifying the design, cohort, and intervention characteristics. The revised text now reads: “This study is a secondary analysis of a randomized controlled trial in which healthy older men completed 16 weeks of HReT performed three times per week.”

The statement “response variability is widely assumed to reflect true inter-individual variability” may be seen as a rhetorical overgeneralization, since part of the scientific community is already aware of the statistical limitations involved, and many recent studies have applied appropriate analytical approaches (e.g., mixed models, typical error thresholds). A more balanced phrasing is recommended, such as:
“Although several studies assume… this assumption is not always supported by rigorous statistical evaluation.”

We agree with the reviewer that the original phrasing could be interpreted as an overgeneralization. The sentence has been amended to provide a more balanced and precise formulation. The revised text now reads: “Although several studies have interpreted this response variability as evidence of true inter-individual variability, this assumption is not always supported by rigorous statistical evaluation (Senn, 2016).”

2. Methods
2.1. Study Design, and Setting
The authors state that there were no differences between the losartan and placebo groups on outcomes of muscle mass and strength, and therefore merged them into a single exercise group for the current analysis. However, this rationale may be insufficient without reporting the statistical power of the original comparison or the magnitude and precision of the between-group differences (e.g., effect sizes, confidence intervals). A non-significant result does not necessarily imply equivalence, especially if the original study was underpowered to detect meaningful differences. Providing such information would help justify the decision to pool the groups and ensure that the conclusions of the secondary analysis are not biased by an unrecognized pharmacological effect.

We thank the reviewer for this important comment. We fully agree that a non-significant result does not in itself imply equivalence, and that presenting the magnitude and precision of between-group differences is critical for transparency.

The original randomized controlled trial was powered to detect clinically meaningful effects of losartan on muscular outcomes, but not specifically on differences in inter-individual variability. Given that the primary analyses showed no meaningful differences between the losartan and placebo exercise groups, we pooled them in the present secondary analysis to maximize statistical power and improve the precision of SDIR estimates.

To address this point, we have now included Supplemental Table 2, which provides summary statistics, effect sizes, and 95% confidence intervals for between-group differences (LOS-EX vs. PLA-EX) for all primary outcomes (MVC, RFD, qCSA, type I and II fCSA, and 1RM). These data confirm that no statistically significant or meaningful differences were present, supporting the appropriateness of pooling the two exercise groups for the variability analyses.

2.2. Study Population and 2.3. Randomization
The inclusion and exclusion criteria are well defined, ensuring a relatively homogeneous and healthy older male cohort. The use of stratified block randomization based on physiologically relevant variables (thigh lean mass, ACE genotype, age) strengthens internal validity. However, the exclusive inclusion of males limits the generalizability of findings, and this should be acknowledged in the discussion.
More critically, while the authors combined the two exercise groups (losartan and placebo) for the current analysis, no justification regarding the statistical power of the original comparison is provided. A post hoc equivalence analysis or at least a summary of the between-group results with confidence intervals would be needed to validate this decision. Furthermore, the absence of a true placebo + sedentary group confirms that the study lacked a fully blinded control group without intervention, which should be acknowledged as a methodological limitation.

We thank the reviewer for raising these important points. Regarding statistical power, as noted above, the original trial was powered to detect clinically meaningful effects of losartan on muscular outcomes, not on differences in inter-individual variability. Since no meaningful between-group differences were observed, pooling the losartan and placebo exercise groups allowed us to increase power and precision in the current secondary analysis. The between-group effect sizes and confidence intervals are now presented in Supplemental Table 2, supporting this decision.

In addition, we have added a clear statement to the Limitations section acknowledging that pooling groups could mask subtle drug effects that the study was not powered to detect. We have also explicitly noted the absence of a true control group without either exercise or losartan intervention, which may limit the ability to fully isolate training effects from other influences.

- “Second, the analysis pooled participants who received either losartan or placebo. Although previous work from our group has shown no effect of losartan on muscle maintenance or neural innervation (Heisterberg et al., 2018; Soendenbroe et al., 2022), we re-calculated SDIR values including only participants who received losartan, and the results were unchanged. Nonetheless, we cannot fully exclude the possibility that subtle effects were not detected.”
- “Third, the study did not include a true control group without either exercise or losartan treatment, which may have limited the ability to fully isolate training effects from other influences.”

Finally, we have reiterated that the exclusive inclusion of healthy older men constrains the generalizability of the findings to broader populations: “Fourth, the study included only healthy older men, and no women or frail individuals were recruited.”

2.4. Intervention
The resistance training intervention (HReT) is generally well described, with clearly defined duration, frequency, supervised sessions, and progressive intensity based on repeated 1RM testing. These features enhance the internal validity of the study. However, the structure of the six training phases is only briefly mentioned and would benefit from greater detail (e.g., duration, weekly progression, and set/rep schemes) to ensure replicability and allow proper quantification of training volume.
Additionally, no data on training adherence are reported, which are crucial for interpreting inter-individual variability. Differentiating between poor responders and poor compliers requires at least basic adherence metrics (e.g., number of sessions attended). Including this information would strengthen the interpretation of the findings.

We fully agree with the reviewer’s point and have expanded the description of the training intervention to include more detailed information on the six training phases, including progression of sets, repetitions, and relative training intensities. The revised section now reads: “For the leg press and leg extension exercises, the training program consisted of six distinct phases that systematically increased training intensity and reduced the number of repetitions: 3 × 12 at 15 RM (Phase 1), 4 × 10 at 12 RM (Phase 2), 4–5 × 8–10 at 10 RM (Phase 3), 5 × 6–10 at 8–10 RM (Phase 4), 4–5 × 6–8 at 8 RM (Phase 5), and 4 × 4–8 at 6–8 RM (Phase 6). Minor differences between the two exercises were present. The leg curl followed a similar structured progression but with slightly fewer sets.”

Regarding adherence, participants were required to complete the full training program before post-testing. The average number of completed training sessions was 48 (range 44–53), reflecting minor variation in the total duration of the intervention due to scheduling issues.

2.6. Data Analysis
The outcome measures used in the study are comprehensive and well selected, spanning morphological (qCSA, type II fCSA), functional (MVC, RFD), and histological domains. These were assessed using established gold-standard methodologies, and procedures appear to have been applied with consistency and blinding, which enhances the internal validity of the measurements. Furthermore, the analytical framework employed to characterize interindividual variability is conceptually robust. The authors adopt a widely accepted approach that includes the calculation of SDIR to detect net individual variation beyond random noise, as well as the use of typical error (TE) to classify individual responsiveness. The integration of multiple outcome domains into a composite responder classification provides a broader representation of adaptation and reflects current trends in the field.
However, some critical limitations must be acknowledged. First, the manuscript does not report the measurement reliability parameters, such as TE and coefficient of variation (CV), that are essential to support the interpretation of response classifications. Without these, the accuracy of thresholds used to determine positive, negative, or trivial responses is uncertain.

We appreciate the reviewer’s constructive feedback. In response, we have added the typical error (TE) values for all relevant outcome measures in the Results section to support the interpretation of the responder classifications and enhance transparency regarding measurement reliability.

Specifically, we have included the following statement: “The TE values used to classify individual responsiveness were: MVC = 10 Nm, RFD = 277 Nm/s, qCSA = 1.25 cm², type I fCSA = 562 µm², and type II fCSA = 582 µm² (Table 3).”

Second, the discretization of individual responses into +1, 0, and –1 categories for each outcome, while practical for visualization, may oversimplify the true biological variability and reduce interpretability.

We agree with the reviewer that categorizing responses as Poor, Trivial, Robust, and Excellent is based on arbitrary thresholding, and that alternative thresholds would likely yield different classifications. We also recognize that this discretization may simplify the underlying biological variability. However, we believe that this simple and transparent classification approach has practical value, as it allows integration of multiple outcome measures into a single, interpretable framework. A more granular system (e.g., with multiple gradations of “poor” or “excellent”) could offer finer resolution but would also introduce additional complexity and reduce interpretability.

To acknowledge this important point, we have explicitly noted this as a limitation in the revised manuscript: “Sixth, the classification of individuals into Poor, Trivial, Robust, and Excellent responders was based on a specific thresholding approach (typical error). While widely used, this approach is inherently arbitrary, and different thresholds or statistical frameworks (e.g., SWC) could yield different classifications.”

Third, the analysis would be strengthened by the inclusion of formal statistical tests for heterogeneity of variance (e.g., Levene’s test) to complement the descriptive SDIR approach.

In line with the comment, we conducted formal tests of heterogeneity of variance (F-tests) for all 10 outcome–timepoint combinations. Nine tests were non-significant, and one reached p < 0.05 (qCSA 8 wk), which is expected by chance at this number of comparisons. This single result did not remain significant after correction for multiple testing. While we agree that such formal tests can provide complementary information, our primary analytic strategy follows the established approach in exercise physiology for evaluating inter-individual variability [9,10] - using SDIR estimation and TE-based classification to separate biological variability from measurement noise. It should also be noted that formal tests for variance equality are often underpowered in modest samples, and their results must therefore be interpreted with caution in this context.

Most importantly, a fundamental design limitation restricts the validity of interindividual inferences drawn from the data. The comparison of variability in training response is conducted between two distinct groups (SED vs. EX), each comprising different participants. Such a between-subjects design is inherently vulnerable to confounding factors, including differences in genetic background, biological rhythms, habitual activity, nutrition, and other individual characteristics that may influence the outcomes independently of the intervention. This undermines the ability to isolate true interindividual response variability to the exercise intervention itself. As previously proposed in the literature (Chaves et al., 2025; PMID: 39958513), within-subject designs, where one limb serves as control and the contralateral limb receives the experimental stimulus, provide a more rigorous alternative. These designs inherently control for between-subject biological variation and shared systemic influences such as hormonal fluctuations, sleep, and dietary intake, thereby offering superior sensitivity to detect true variability in responsiveness. It is recommended that the authors explicitly acknowledge, in the Discussion or Limitations section, the implications of employing a between-subject design for interpreting inter-individual variability. A brief mention of alternative approaches, such as within-subject or contralateral limb designs, would enhance the manuscript’s conceptual depth and demonstrate awareness of contemporary methodological advances in the study of individual responsiveness to resistance training.

We fully agree with the reviewer that the use of a between-subject design introduces potential sources of biological confounding that may influence the estimation of inter-individual variability. It is worth noting that this study was originally designed to evaluate the effect of a systemically acting pharmacological agent (losartan), which necessitated a between-subject design. We also acknowledge that within-subject or contralateral limb designs [11] offer a powerful alternative to control for shared systemic influences such as hormonal fluctuations, nutrition, and sleep. At the same time, these designs have their own limitations, including cross-education effects on strength outcomes [12], which may complicate interpretation.

We have therefore added a paragraph in the Discussion acknowledging these methodological trade-offs and referencing alternative study designs: “Emerging methodological approaches, such as within-subject or contralateral limb designs [11], offer powerful ways to control for systemic sources of variability, though these also come with trade-offs such as potential cross-education effects [12].”

3. Results
The results section presents individual-level outcomes across multiple domains of muscle adaptation (fCSA, qCSA, MVC, RFD, 1RM), allowing for an integrated view of training responsiveness. The use of individual classification plots and heatmaps is visually effective and aligns with the study’s stated aim of exploring inter-individual variability. The inclusion of a composite responsiveness score adds analytical depth and facilitates the identification of distinct responder subgroups. However, several issues warrant consideration:
First, despite the richness of the dataset, the results are presented in a primarily descriptive fashion. No inferential statistics are used to compare the losartan and placebo groups, nor are formal variance analyses conducted to support the interpretation of inter-individual heterogeneity. This absence weakens the ability to distinguish whether observed differences across participants reflect meaningful biological variability or statistical noise.

As noted in our response to the previous comment, our primary analytical focus was on descriptive methods (individual-level plots, heatmaps, and composite responsiveness scores), which align with the standard analytical approach used in previous exercise training studies examining variability in adaptation [9,10].

To enhance transparency, we have now provided group-level variability data for each treatment arm separately. Specifically, SD_LOS-EX_ and SD_PLA-EX_ are shown in Supplemental Table 1, while SD_SED_ and SD_EX_ (pooled exercise groups) remain in Table 3. In addition, we have included a Supplemental Table 2, which summarizes group-level changes in all key outcomes for LOS-EX and PLA-EX. These data confirm that there were no statistically significant or meaningful differences between the two exercise groups, supporting our decision to combine them for the main analyses.

Second, although measurements were conducted at three time points (PRE, 8wk, and 16wk), the results focus exclusively on baseline to post-training (PRE–16wk) changes. The omission of temporal trajectories for individual participants is a missed opportunity to explore nonlinear or early adaptive patterns, which could enhance mechanistic understanding.

We appreciate the reviewer’s point and agree that temporal trajectories can provide valuable insight into the timing and pattern of individual adaptations. As noted, we do include comparisons between baseline and 8 weeks in Figures 1 and 2, thereby capturing part of the temporal dimension. However, subsequent analyses (e.g., 1RM progression and baseline level associations) focused on baseline to 16 weeks. This was primarily done for simplicity, but also because some studies have shown leg-differences in morphometric outcomes, such as fiber size and proportions.

While we agree that more detailed temporal analyses could be informative, this falls outside the primary aim of the present manuscript, which was to determine the existence of inter-individual variability in adaptation to HReT. As the reviewer notes, meaningful exploration of temporal trajectories would ideally be paired with mechanistic assessments (e.g., neuromuscular, tendon, and bone adaptations), which we view as an important avenue for future work.

Third, while the heatmap-based visualization of +1/0/–1 scores allows for intuitive interpretation, the final classification of participants into five responsiveness categories (e.g., overall responders, non-responders, etc.) appears somewhat arbitrary and is not supported by cluster analysis or other multivariate techniques. Furthermore, it is unclear how robust these classifications are to variations in the composite score threshold.

We agree with the reviewer that the classification framework is inherently arbitrary, and that different thresholding or analytical strategies could produce different categorizations. However, to our knowledge, no equivalent classification approach has previously been applied to HReT, and we view this as a transparent and pragmatic framework that can be further refined and built upon in future work. Importantly, for a response to be counted as positive or negative, it must exceed the pre-defined TE, which helps reduce the number of spurious classifications.

To acknowledge this explicitly, we have added the following statement to the Limitations section: “Sixth, the classification of individuals into Poor, Trivial, Robust, and Excellent responders was based on a specific thresholding approach (typical error). While widely used, this approach is inherently arbitrary, and different thresholds or statistical frameworks (e.g., SWC) could yield different classifications.”

Finally, the decision to pool participants across treatment groups without presenting between-group analyses introduces ambiguity. Although a rationale for this choice is discussed elsewhere, it limits interpretation, especially if subtle treatment effects were present but underpowered to reach significance. It would be helpful to report group-level variability (e.g., SDEX) separately for each treatment arm.

We thank the reviewer for this important comment. As noted, the primary rationale for pooling the losartan and placebo exercise groups was that the original randomized controlled trial showed no meaningful differences between groups on the key outcomes, and the present secondary analysis focuses on inter-individual variability in training response, not on drug effects per se. Pooling the exercise groups therefore maximizes statistical power and improves the precision of SDIR estimates.

To enhance transparency, we have now provided group-level variability data for each treatment arm separately. Specifically, SD_LOS-EX_ and SD_PLA-EX_ are shown in Supplemental Table 1, while SD_SED_ and SD_EX_ (pooled exercise groups) remain in Table 3.

In addition, we have included a Supplemental Table 2, which summarizes group-level changes in all key outcomes for LOS-EX and PLA-EX. These data confirm that there were no statistically significant or meaningful differences between the two exercise groups, supporting our decision to combine them for the main analyses.

4. Discussion
The discussion is generally well-written and grounded in the broader scientific literature. The authors clearly articulate the relevance of investigating inter-individual variability in response to RT, particularly in older adults, and contextualize their findings in light of past studies. They appropriately acknowledge prior concerns about misinterpreting individual responses, and adopt a cautious and technically justified approach by incorporating TE thresholds and composite scores. The observation that only two individuals showed limited benefit across four muscle outcomes supports their argument that “true non-responders” may be rare, especially when outcome measures are comprehensive. The authors are also commended for avoiding the term “non-responder” and for discussing the limitations of task-specific improvements such as 1RM. However, a few important considerations should be noted:
First, the interpretation of responders vs. non-responders depends heavily on the reliability of the measurements used. Although the TE approach is methodologically sound, the authors do not report test-retest reliability indices (e.g., TE and, CV) from their own dataset. These are critical to justify the thresholds used for response classification.

We agree with the reviewer that the interpretation of responder classifications relies on the reliability of the underlying measurements. In response, we have added TE values to the Results section to aid in the interpretation of individual responses. These values were calculated from the SED group between PRE–8 wk and PRE–16 wk, consistent with standard approaches in the field. We acknowledge that we do not have repeated measures from the same testing session, which would have provided additional precision.

Second, the observed discrepancy between gains in MVC and fCSA highlights the limitation of assuming parallel adaptation across structural and functional domains. While the multidimensional framework adopted is commendable, the decision to exclude 1RM due to its learning component could be debated, especially since 1RM improvements may carry significant clinical relevance.

We agree with the reviewer that structural and functional outcomes do not necessarily follow the same time course, and we have expanded the Discussion to explicitly highlight this point: “Neural adaptations, such as improved motor unit recruitment and firing efficiency, typically manifest earlier, whereas hypertrophic processes begin from the first training sessions but only become detectable later.”

We also agree that 1RM improvements are important, and we have expanded this to: “…indicating that, in practical terms, the training was effective and that 1RM improvements likely reflect meaningful functional gains with clinical relevance.”

However, 1RM was not included in the responder classification for several methodological reasons. The test was performed in a less controlled setting than MVC testing (e.g., different testers, testing in a private gym environment with potential distractions, and rounding to the nearest 2.5 kg), and is also influenced by a substantial learning effect, particularly in untrained older adults. Collectively, these factors reduce the measurement precision of 1RM compared to MVC and fCSA, making it less suitable for use in a TE-based responder classification framework.

Third, the identification of poor responders based on fCSA or qCSA reductions, particularly in cases where technical error is suspected, underscores the importance of assessing data quality and accounting for sources of error, even when using control groups.

We fully agree with the reviewer that careful assessment of data quality and potential technical error is essential, even when control groups are included. In our study, the most pronounced outlier occurred in qCSA, where one participant showed an implausible 5% reduction over 16 weeks, which almost certainly reflects measurement error rather than a true biological response. This specific case is already highlighted in the Discussion to illustrate the importance of critically evaluating such data, and we therefore have not made further changes to the text.

Finally, and importantly, the longitudinal design of the study, with measurements at baseline, 8 weeks, and 16 weeks, provides an excellent opportunity to explore dynamic response trajectories. Although the authors note that the proportion of Poor/Trivial responders decreased from 32% to 18% over time, they do not analyze or discuss whether some participants may be classified as early, late, or sustained responders. This is a missed opportunity. A more detailed analysis of temporal response patterns could shed light on the variability in adaptation kinetics among older adults and offer practical implications for training duration and progression strategies. Classifying individuals by response trajectory, or presenting individual spaghetti plots, would have enriched the interpretation and could inform more personalized exercise interventions.
We agree that temporal response trajectories can provide valuable insights into adaptation kinetics and individual variability. However, a detailed trajectory analysis was not the primary aim of the present work, which focused on establishing the existence of inter-individual variability in response to HReT. We therefore chose to limit the analyses to baseline–8wk and baseline–16wk, which already capture part of the temporal dimension, while keeping the manuscript focused and interpretable.

To acknowledge this point, we have added the following statement to the Discussion:
“Given that nearly all participants demonstrated early strength gains, responder classification after 8 weeks largely depends on whether these functional improvements were accompanied by measurable hypertrophy, which typically develops on a slower time course.

We agree that more detailed classification of temporal response patterns represents an important avenue for future research.

5. Conclusions
The conclusion is concise and aligns with the main findings of the study. The authors appropriately reiterate that high-load resistance training (HReT) effectively increases muscle mass and strength at the group level and that individual responses show variability. The assertion that true non-responders are rare is supported by their two-pronged analytical approach and the use of multiple outcome domains with TE-based classification. However, the conclusion could be further strengthened by integrating some of the key nuances discussed earlier in the manuscript. Specifically, while the rarity of non-responders is a central takeaway, it is important to emphasize that this conclusion depends on the comprehensiveness of the outcome measures used, the robustness of the measurement protocols, and the choice of statistical thresholds.

We agree with the reviewer that the conclusion should more explicitly reflect the analytical framework underlying this interpretation. We have therefore revised the conclusion to incorporate this nuance. The revised text now reads:
“HReT is effective at increasing muscle mass and strength at the group level, yet individual responses vary considerably. When multiple outcome domains (muscle mass and strength) and typical error–based thresholds are applied, true non-responders appear rare among healthy older men. HReT should remain the universally recommended first-line strategy for increasing muscle mass and strength in older adults.”

Additionally, the finding that certain individuals required more time (i.e., responded only by 16 weeks) underscores the importance of training duration and the potential presence of early versus late responders. These temporal aspects are not acknowledged in the final paragraph, despite being critical for tailoring interventions in older populations.

We appreciate the reviewer’s insightful observation regarding temporal response patterns. We have revised the text to explicitly acknowledge this point, adding the following sentence to the Discussion section: “This suggests that a longer training duration may be necessary for some older adults to fully realize the benefits of HReT, highlighting the presence of early and late responders and the importance of maintaining training over time.”

This addition emphasizes the temporal dimension of responsiveness, which may have important implications for how training programs are structured for older adults.

Lastly, the recommendation for HReT as a universally applicable strategy is well justified in light of the data but should be cautiously interpreted in light of participant characteristics (i.e., healthy older men) and potential challenges in generalizing findings to more heterogeneous or frail populations. Including this caveat would enhance the external validity of the concluding statement.

We appreciate the reviewer’s important point regarding the generalizability of our findings. We have revised the Conclusion to explicitly state that the recommendation for HReT as a first-line strategy applies to this specific population of healthy older men, and we have also added a statement to the Limitations section noting the absence of women and frail individuals in the study cohort. This ensures that the findings are appropriately contextualized.

**References**

1	McKay AKA, Stellingwerff T, Smith ES, *et al.* Defining Training and Performance Caliber: A Participant Classification Framework. *Int J Sports Physiol Perform*. 2022;17:317–31. doi: 10.1123/ijspp.2021-0451

2	Benito PJ, Cupeiro R, Ramos-Campo DJ, *et al.* A Systematic Review with Meta-Analysis of the Effect of Resistance Training on Whole-Body Muscle Growth in Healthy Adult Males. *Int J Environ Res Public Health*. 2020;17:1285. doi: 10.3390/ijerph17041285

3	Hubal MJ, Gordish-Dressman H, Thompson PD, *et al.* Variability in muscle size and strength gain after unilateral resistance training. *Med Sci Sports Exerc*. 2005;37:964–72.

4	Ahtiainen JP, Walker S, Peltonen H, *et al.* Heterogeneity in resistance training-induced muscle strength and mass responses in men and women of different ages. *Age (Dordr)*. 2016;38:10. doi: 10.1007/s11357-015-9870-1

5	Churchward-Venne TA, Tieland M, Verdijk LB, *et al.* There Are No Nonresponders to Resistance-Type Exercise Training in Older Men and Women. *J Am Med Dir Assoc*. 2015;16:400–11. doi: 10.1016/j.jamda.2015.01.071

6	Heisterberg MF, Andersen JL, Schjerling P, *et al.* Losartan has no additive effect on the response to heavy-resistance exercise in human elderly skeletal muscle. *J Appl Physiol (1985)*. 2018;125:1536–54. doi: 10.1152/japplphysiol.00106.2018

7	Soendenbroe C, Heisterberg MF, Schjerling P, *et al.* Human skeletal muscle acetylcholine receptor gene expression in elderly males performing heavy resistance exercise. *Am J Physiol Cell Physiol*. 2022;323:C159–69. doi: 10.1152/ajpcell.00365.2021

8	Soendenbroe C, Svensson RB, Mittendorfer B, *et al.* Morphological differences in myofibre size and shape: A comparative study of the soleus, gastrocnemius, triceps brachii and vastus lateralis in humans and mice. *J Anat*. Published Online First: 17 July 2025. doi: 10.1111/joa.70025

9	Walsh JJ, Bonafiglia JT, Goldfield GS, *et al.* Interindividual variability and individual responses to exercise training in adolescents with obesity. *Appl Physiol Nutr Metab*. 2020;45:45–54. doi: 10.1139/apnm-2019-0088

10	Cano-Montoya J, Rojas Vargas M, Báez Vargas S, *et al.* Impact of resistance and high-intensity interval training on body composition, physical function, and temporal dynamics of adaptation in older women with impaired cardiometabolic health: a randomized clinical trial. *BMC Sports Sci Med Rehabil*. 2025;17:78. doi: 10.1186/s13102-025-01119-0

11	Chaves TS, da Silva DG, Lixandrão ME, *et al.* Within-individual design for assessing true individual responses in resistance training-induced muscle hypertrophy. *Front Sports Act Living*. 2025;7. doi: 10.3389/fspor.2025.1517190

12	Calvert GHM, Carson RG. Neural mechanisms mediating cross education: With additional considerations for the ageing brain. *Neurosci Biobehav Rev*. 2022;132:260–88. doi: 10.1016/j.neubiorev.2021.11.025
